# Association Mechanism of *S*-Dinitrophenyl Glutathione with Two Glutathione Peroxidase Mimics: 2, 2′-Ditelluro- and 2, 2′-Diseleno-bridged β-cyclodextrins

**DOI:** 10.3390/molecules14030904

**Published:** 2009-02-25

**Authors:** Ya-Qiong Hao, Xing-Chen Liu, Jun-Qiu Liu, Yu-Qing Wu

**Affiliations:** State Key Laboratory for Supramolecular Structure and Materials, Jilin University, Changchun, 130012, P. R. China; E-mails: yqhao2003@yahoo.com.cn (Y-Q. H.), xingchen.liu@hotmail.com (X-C. L.), junqiuliu@jlu.edu.cn (J-Q. L.)

**Keywords:** 2,2′-Ditelluro-bridged β-cyclodextrins, 2,2′-Diseleno-bridged β-cyclodextrins, Association mechanism, MM2 modeling, NMR spectroscopy

## Abstract

Complex formation of the glutathione peroxidase mimics 2,2′-ditelluro-bridged β-cyclodextrin (**1**) and 2,2′-diseleno-bridged β-cyclodextrin (**2**), with *S*-substituted dinitrophenyl glutathione (**3**) were determined by ultraviolet-visible (UV-Vis) absorption spectroscopy in phosphate buffer (pH 7.4) and ^1^H-NMR spectroscopy. Molecular mechanics (MM2) modeling calculations were used to deduce a three-dimensional model for each complex. The dinitrophenyl (DNP) group of **3** appears to penetrate the cavity of β-cyclodextrin (β-CD) or **1**, but it is located between the two secondary rims of **2**. The complexes’ stability constants (*K*_s_) from 19 to 37 °C, Gibbs free energy changes (*ΔG°*), *ΔH°* and T*ΔS°* for 1:1 complexes of β-CD, **1** and **2** with ligand **3** as obtained from UV-Vis spectra were compared. The binding of **3** by the three cyclodextrin hosts generally decreased in the order of **1**>**2**>β-CD. The binding ability of **3** by β-CD, **1** and **2** was discussed with regard to the size/shape-fit concept, the induced-fit interaction, and the cooperative interaction of the dual hydrophobic cavities. The binding ability of **1**>**2** indicated that the length of linkage between two cyclodextrin units plays a crucial role in the interaction with **3**.

## Introduction

In the fields of Enzymology and Biology, considerable efforts have been applied recently to the synthesis of compounds that mimic the properties of glutathione peroxidase (GPX) in catalyzing the reduction of hydrogen peroxide. Such artificial compounds, which have the potential to become enzyme therapy agents, are designed to overcome the limitations associated with the authentic enzyme [1,2]. Wilson and coworkers synthesized some diselenides and suggested that the diselenoyl bond should be used as a catalytic moiety to imitate GPX [3]. Tellurium and selenium exhibit similar redox properties and thus some diorganyl tellurides have been prepared to mimic the catalytic properties of GPX [4,5,6,7,8,9], but these investigators did not consider the substrate binding ability of an enzyme model. The substrate binding properties generally play as vital a role in enzymatic function, as does catalytic ability [10]. In this respect, cyclodextrins are an attractive class of materials. They may bind substrates and then either catalyze their reactions or mimic a step in an enzymatic catalytic sequence [11]. 

**Scheme 1:**
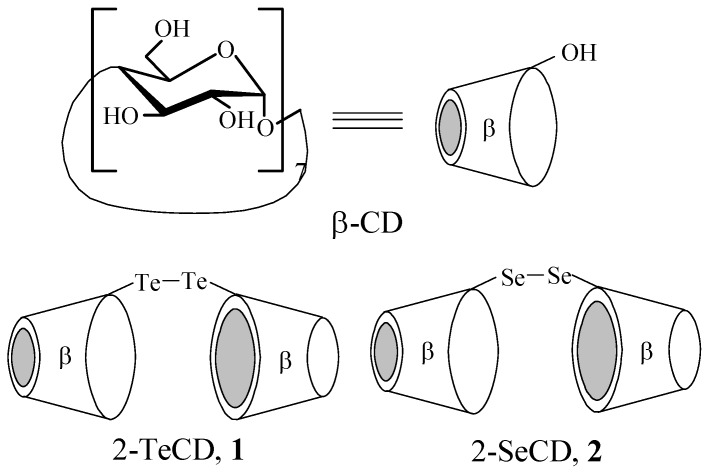
Chemical structures of β-cyclodextrin (β-CD), 2,2′-tellurium-bridged β-cyclodextrin (**1**) and 2,2′-diseleno-bis-β-cyclodextrin (**2**).

**Scheme 2:**
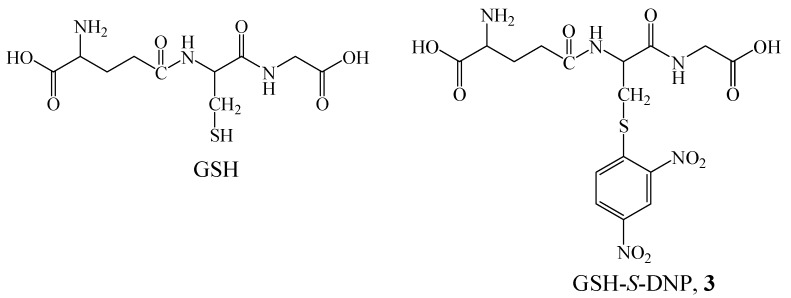
Chemical structures of glutathione (GSH) and *S*-substituted dinitrophenyl glutathione (**3**).

Bridged cyclodextrin dimers have been demonstrated to have greatly enhanced molecular binding ability, as compared to the original parent cyclodextrins due to the cooperative binding of guest molecules in the closely located cavities of cyclodextrin [12,13,14,15,16]. In addition, cyclodextrin dimers provide an excellent model system for mimicking the substrate-specific interaction of GPX enzymes, and therefore are also potential antioxidants in biological systems [17,18]. Recently, a novel GPX mimic, 2,2′-ditelluro-bridged β-cyclodextrin (1) (Scheme 1), has been synthesized [19,20] that catalyzes the reduction of hydroperoxides by glutathione (GSH) (Scheme 2) more efficiently than other GPX mimics such as Ebselen [3] and 2,2′-diseleno-bridged β-cyclodextrin (**2**) (Scheme 1) [21]. 

According to Ren *et al*. [19], under the same conditions, the initial rate of reduction of H_2_O_2_ (0.5 mM) by GSH (1 mM) in the presence of **1** (1.2 μM) is at least seven times more efficient than with **2** and 46 times greater than with Ebselen (1.2 μM). In addition to the high GPX activity, the greater water solubility of **1** has improved its potential in pharmacological applications. Recently, it was proposed that the strong catalytic efficiency of cyclodextrin-derived tellurium may be partly due to the nature of its intermediate state which is different from that of cyclodextrin-derived selenium during the catalytic processes [4,19,20]. However, the mechanism of interaction of the substrate (GSH) with **1** has not yet been investigated substantially. 

To investigate the nature of the difference in GPX activity, we determined the molecular association behavior of **1** in binding with substrate and also compared the substrate-binding properties of **1** and **2**. In consideration of the potential anti-oxidation activity, it is interesting and important to elucidate the binding model between these cyclodextrins and their substrates. GSH is the most common substrate of **1** and **2**, however, due to its lack of chromophore, the detection of GSH is not convenient. *S*-substituted dinitrophenyl glutathione (**3**) (Scheme 2), the substrate analog of **1** and **2**, was used instead of GSH because the binding properties of **3** can be easily monitored by spectroscopic methods. Complex formation was assessed by ^1^H-NMR and ultraviolet-visible (UV-Vis) spectroscopy. The three-dimensional geometry structures of the complexes were deduced from molecular mechanics (MM2) modeling calculations. The stability constants (log *K*_s_) and Gibbs free energy changes (*ΔG°*) of the complexes were obtained for the native β-cyclodextrin (β-CD), **1** and **2** (Scheme 1).

## Results and Discussion

### Spectral titration and thermodynamic measurements

**Figure 1:**
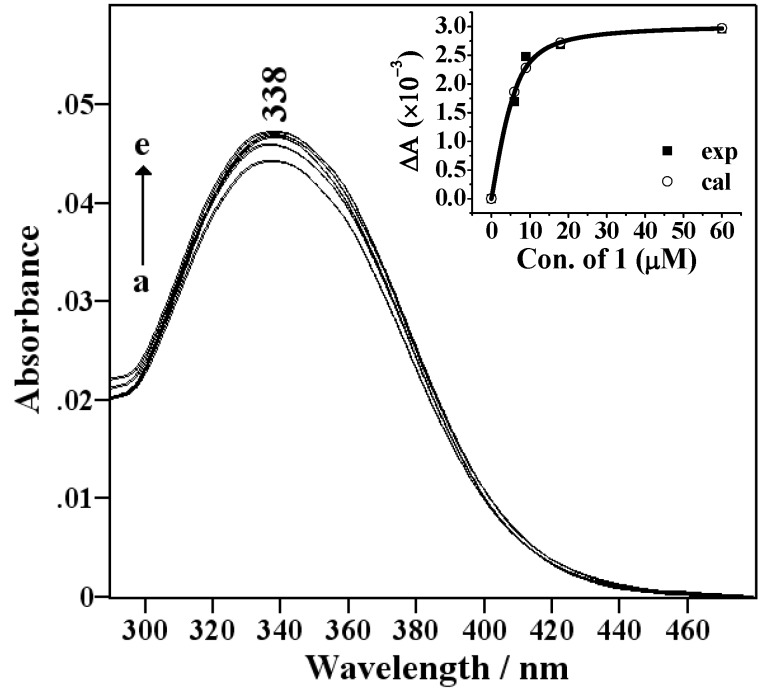
UV-Vis spectra of **3** in a phosphate buffer solution (6 μM) in the presence or absence of **1**. The concentrations of **1** (from a to e) are 0, 6, 9, 18 and 60 μM, respectively. Least-squares curve-fitting analysis (Inset) for the complexation of **3** with **1**.

To determine the stability constant (*K*_s_), UV-Vis titration spectral measurements of **3** in the presence of increasing concentration of **1** were performed at 32 °C. Addition of a known amount of **1** to a dilute solution (6 μM) of **3** caused an enhancement of the UV-Vis absorbance at 338 nm, indicating that the inclusion complex was formed between **1** and **3** (Figure 1).

Assuming a 1:1 stoichiometry as the two cyclodextrin moieties in **1** are treated as a host unit, the

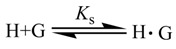
(eq. 1)
complexation of guest (G) with cyclodextrin host (H) is expressed by eq. 1:

Then, the effective stability constant (*K*_s_) [22] can be obtained from changes of absorbance intensity (*ΔA*) at 338 nm with various host concentrations by using a nonlinear least-squares method according to a fitting eq. 2 [23,24], where [H]_0_ and [G]_0_ indicate the total concentrations of **1** and **3**, respectively, and Δε means the differential molar extinction coefficient of **3** in the absence and presence of **1**. For each host-guest combination examined, the inset plot (in Figure 1) of observed *ΔA* (small dots) as a function of the initial host concentration [*H*]_0_ gave an excellent fit to the theoretical value (open circle), verifying the validity of the 1:1 complex stoichiometry assumed above. In the repeated measurements, the *K*_s_ values are reproducible within an error of ±5%.

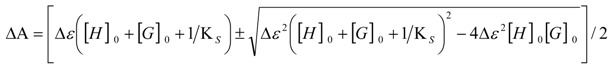
(*eq.* 2)

The stability constants (*K*_s_) between **3** and β-CD alone, or **2** were also performed at the same experimental conditions by using UV-Vis titration spectral measurements. For each cyclodextrin, the *K*_s_ and *Δε* values were obtained (Table 1). 

The *K*_s_ values in Table 1 illustrates clearly that **1** and **2**, both possessing two hydrophobic cyclodextrin cavities with cooperative binding, form a more stable complex with **3** than does β-CD alone. The enhanced binding of **3**, as indicated by the magnitude of *K*_s_ (X)/*K*_s_ (β-CD), is distinctly greater for **1** (4.46) than **2** (1.48).

**Table 1:** Complex stability constant (*K*_s_) for 1:1 inclusion complexation of **3** with β-CD, **1** and **2** in the aqueous buffer solution (pH 7.4) at 32 °C.

Hosts	*K*_s_ [M^-1^]	*K*_s_(X)/*K*_s_(β-CD)^*^	log *K*_s_	*Δε*
β-CD	1.49×10^4^	≡1	4.17	1050
**1**	6.64×10^4^	4.46	4.82	510
**2**	2.21×10^4^	1.48	4.34	960

^*^Relative selectivity for *S*-substituted dinitrophenyl glutathione (**3**).

**Figure 2:**
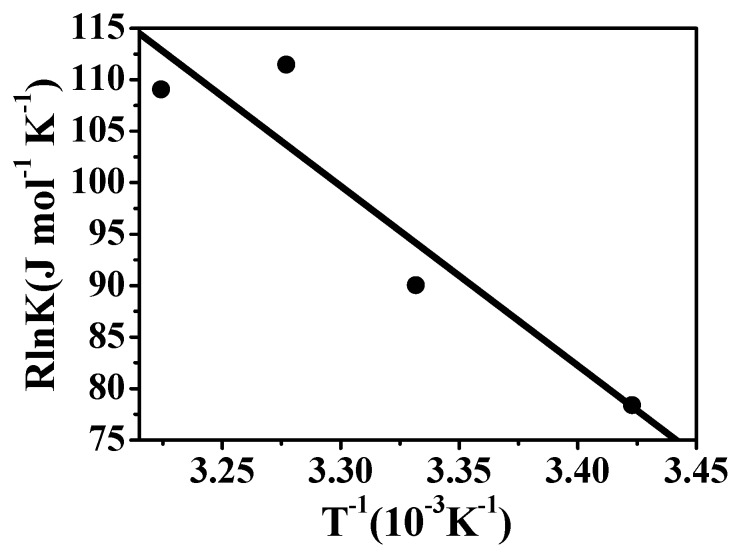
Van’t Hoff plots for the associations of **3** and **1** from UV-Vis results.

To obtain thermodynamic parameters, UV-Vis titration spectral measurements of **3** with β-CD, **1** or **2** were performed at several temperatures ranging from 19 to 37 °C. The complex stability constants (*K*_s_) at different temperatures were calculated and the corresponding van’t Hoff plot was prepared (Figure 2). If *ΔCp°* =0, the experimental values for *RlnK_s_* fit the well-known linear equation, eq. 3:

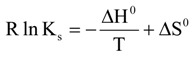
(eq. 3)

*ΔH°* and *ΔS°*, which are temperature independent, can be estimated from the slope and intercept of the fit plot, respectively. The Gibbs free energy changes (*ΔG°*) can be obtained according to eq*.* 4.

ΔG° = ΔH° − TΔS°
(eq. 4)

The thermodynamic parameters obtained for each inclusion complexation of cyclodextrins with **3** were listed in Table 2.

The formation of complexes of **3** and the cyclodextrins may be explained as an overall result of the reorganization of solute and solvent molecules after their association. The large positive entropy change is attributed to the extensive desolvation from the hydrophilic moieties of host and ligand and also the relatively high flexibility of the ligand accommodated in the cavity of host [25,26,27,28]. Table 2 illustrates that the formation of complexes are all endothermic processes as indicated by the positive enthalpic changes (*ΔΗ°*). Considering the highly positive entropy changes with less favorable enthalpic changes (|*ΔΗ°* | < |*ΤΔS°* |), we may be able to conclude that formation of complexes are driven by entropy.

**Table 2:** Thermodynamic parameters for the inclusion complexation of **3** with β-CD, **1** and **2** in the aqueous phosphate buffer solution (pH 7.4) at 25 °C.

Hosts	-*ΔG°* [kJ mol^-1^]	*ΔH°* [kJ mol^-1^]	*TΔS°* [kJ mol^-1^]
β-CD	22.095	81.649	103.744
**1**	26.908	174.497	201.404
**2**	22.402	261.049	283.451

### ^1^H-NMR spectroscopy

The ^1^H-NMR spectra of **3** in the presence of different kinds of cyclodextrins were expected to provide information about the binding of cyclodextrin complexes because the induced chemical shifts of **3** observed upon inclusion into cyclodextrin could help to establish an approximate geometry for the complexes. 

**Figure 3:**
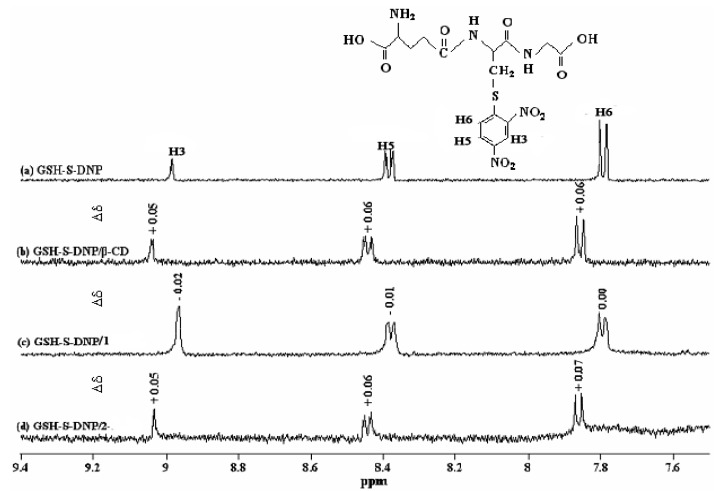
^1^H-NMR spectrum of **3** in the absence (a) and in the presence of (b) β-CD, (c) **1** and (d) **2** in D_2_O at ambient temperature.

The ^1^H-NMR spectra in Figure 3, especially for the aromatic region of the **3**, changed significantly upon addition of 1 equiv. cyclodextrin at ambient temperature. Notably, in this region, the three kinds of cyclodextrin have no signals, and thus provide no interference in the spectra. After the addition of β-CD (**b**, Figure 3) or **2** (**d**, Figure 3), moderate downfield shifts were observed for H3, H5 and H6 protons in comparison to the spectrum of **3** alone (**a**, Figure 3). In contrast, slight upfield shifts of dinitrophenyl (DNP) protons as well as significant lines broadening, especially that of the H3 proton after the addition of **1**, are shown in 3c. However, the obvious wideness of the signals of the outside protons of β-CD in the presence of **3** (data not shown) provided evidence supporting that the interaction of β-CD and **3** did exist in the complexation.

### Molecular mechanics (MM2) modeling calculations

**Figure 4:**
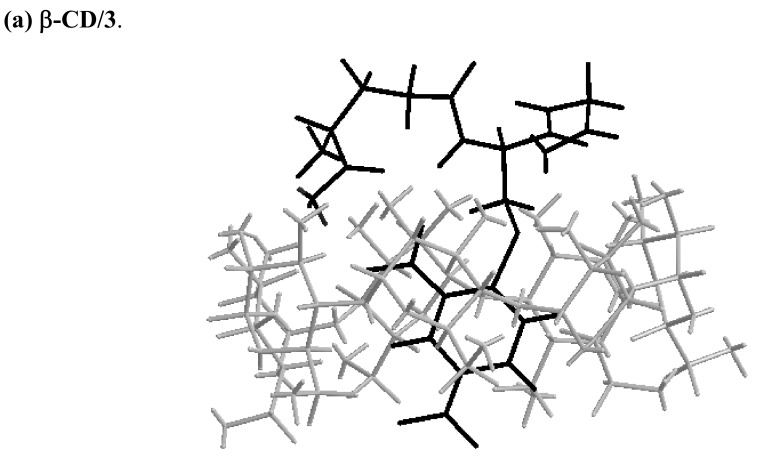

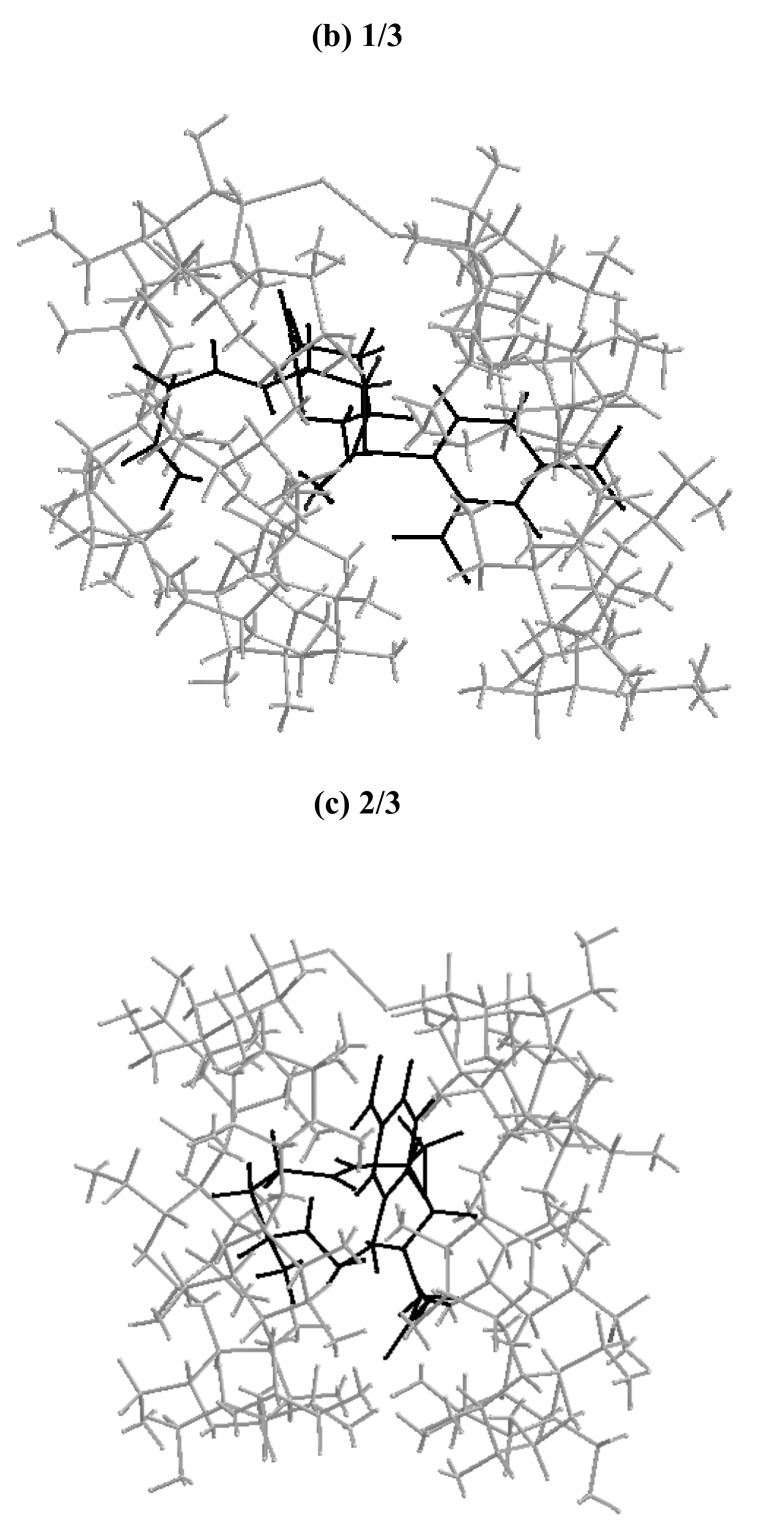
The energy-minimized structure of the complexes of (a) β-CD/**3**, (b) **1**/**3** and (c) **2**/**3**.

To obtain better insight into the complexes formed between **3** and all the cyclodextrins, computational studies on host-guest interactions were carried out to define the most probable conformation of the complexes and the appropriate three-dimensional representation of the complexes. The energy minimum structures of β-CD, **1** or **2**/**3** complexes calculated by Chem3D were shown in Figure 4. Stabilization energy (Δ*E*) upon complexation was calculated for the minimum energy structure according to eq*.* 5 [29,30]:

ΔE = E_complex_ − (E_host_ + E_guest_) (eq. 5)

MM2 calculations show that the preferred orientation of the complexes is that in which the DNP group of **3** is inserted inside the cavity of β-CD (**a**, Figure 4) or **1** (**b**, Figure 4) from the secondary rim, while it situates between the two secondary rims of **2** (**c**, Figure 4) cavities. The structures of the complexes by MM2 calculation also suggest that the long axis of the DNP group is almost parallel to the axis of cyclodextrin cavities in β-CD or **1**, but the DNP axis is tilted at some extent relative to the axis of cavities in **2**. The significant differences between the minimized energies of the complexes that are favored with respect to minimized energies of the two separate molecules indicate a high degree of stability, favoring the formation of complexes. A comparison of the minimized energy among the three complexes reveals an increasing difference energy sequence of β-CD/**3** (70 kcal mol^-1^) < **2**/**3** (75 kcal mol^-1^) < **1**/**3** (106 kcal mol^-1^). That is, **1** and **2** show larger energy difference than native β-CD.

The stability constant (*K*_s_) of **1**/**3** (6.64×10^4^ M^-1^) is slightly higher than that of **2**/**3** (2.21×10^4^ M^-1^) (Table 1), indicating that **3** is bound more strongly with **1** than with **2**. From the thermodynamic data in Table 2, it can be concluded that **1**/**3** forms a more stable complex (*ΔG°* = -26.908 kJ mol^-1^) than is formed for **2**/**3** (*ΔG°* = -22.402 kJ mol^-1^) and β-CD/**3** (*ΔG°* = -22.095 kJ mol^-1^). This stability may be attributed to the linkage length and the relative rigidity of the bridged linkage in **1**, which favors the binding of ligand **3** more than those of **2**. Tellurium, possessing a larger radius and lower electro-negativity than selenium, can provide longer (0.708 nm) and more flexible -Te-Te- bonds than those of -Se-Se- (0.627 nm), which can modify the molecular binding behavior of **1** more efficiently than of **2**. This explanation should be reasonable based on the reported result of disulfide β-CD [15,31]. A study on the binding of a secondary cyclodextrin dimer containing the shorter disulfide linkage (-S-S-, 0.568 nm) showed no enhanced binding ability with a series of substrates because the tight disulfide linkage crowds the system unduly. Therefore, it can be concluded that the involved bridge can function not only as a passive linker but also be a versatile coordinating site that can control the orientation and binding ability/selectivity of cyclodextrin dimers.

In contrast to the binding behavior of the parent β-CD, **1** and **2** enhance the interaction with ligand **3** through the cooperative binding of the two involved cavities. Therefore, the complexation of **1** or **2** with ligand **3** results in stronger hydrophobic interaction and solvent reorganization in comparison with β-CD, leading to the positive entropic changes (*TΔS°*), which are mainly responsible for the overall stability of the complex. Therefore, the cooperative contribution of the two cavities in **1** and **2** is much more pronounced to give an enhancement of binding ability. The association process, which could lead to the exclusion of water molecules from the hydration shell of **3**, is inherently accompanied by entropic gain. Therefore, it is not difficult to understand the positive enthalpic changes (*ΔH°*) and positive *TΔS°* upon complexation with **1** and **2**.

The downfield shifts of the DNP protons found in the ^1^H-NMR spectrum after binding of **3** with β-CD or **2** may be due to moderate water exposure [32] of the protons of the DNP group within the cyclodextrin cavities. The results of MM2 studies indicate that the DNP group penetrates so deeply into the β-CD cavity from the wider secondary side that part of it protrudes from the cavity from the primary side. Therefore, some of the DNP protons are exposed to water, resulting in the downshift of NMR signals. In addition, the Δδs of the protons of DNP group lies between 0.05 and 0.06 for the complex of β-CD/**3** and between 0.05 and 0.07 for **2**/**3**. From these data one can conclude that the water exposure of DNP protons in these two complexes is similar. However, the complexes structures revealed by MM2 calculation demonstrate completely different inclusion forms between β-CD/**3** and **2**/**3**. The results show that the DNP group of **3** is included inside the cavity of β-CD deeply from the secondary rim, however, in **2** it is situated between the two secondary rims of the cavities. Thus, the MM2 calculations distinguish efficiently the differences in the inclusion forms between β-CD/**3** and **2**/**3**, although both show very similar features in ^1^H-NMR spectra. In addition, MM2 can supply information concerning the insertion geometry of both the chromophore and non-chromophore portions of **3** into **2**. The structure obtained by MM2 calculations in Figure 4c shows that the peptide backbone of **3** is included shallowly in a cleft other than the one located between two cavities. In the case of ICD spectral study (result is not shown), only the inclusion geometry of the chromophoric part of **3** can be obtained based on the sign and intensity of the Cotton effects.

Comparison with β-CD and **2**, in the **1**/**3** complex of the protons of the DNP group show observable upfield shifts that indicate restricted movement of the DNP group arising from inclusion into the cavities of **1**. Not so deep as the inclusion of **3** in β-CD cavity, the penetration of the DNP group into the cavities of **1** is of sufficient depth from the secondary rim to be efficiently shielded from attack of the bulk water. This is highly consistent with the results of MM2, which show the DNP group of **3** penetrating into one of the cavities of **1** with the dipole orientation in parallel with the axis of cyclodextrin.

## Conclusions

The present study indicates that three cyclodextrins (β-CD, **1** and **2**) can form stable complexes with glutathione derivative **3** and the stoichiometry for all the complexes is 1:1. Compounds **1** and **2** greatly enhance the binding ability of **3**, in comparison with the parent β-CD, and the complex stability constants of the bis(β-cyclodextrins) **1** and **2** are larger than native β-CD by factors of 4.46 and 1.48, respectively. The effect of **1** is more pronounced than that of **2** due to more suitable length and rigidity of the bridged linkage for **3**. ^1^H-NMR results and MM2 calculations demonstrate that the inclusion forms and depth as well as the degree of exposure to water of the DNP group in β-CD, **1** and **2** are significantly different from each other; it penetrates the cavity of β-CD or **1** from the secondary side *via* its transition moment being parallel to the axis of cyclodextrin cavities; but for **2**, it locates between the two secondary rims of **2** cavities being tilted at some extent to the axis of its cavities. Such differences result from the cooperative interactions of the dual hydrophobic cavities in **1** and **2**, and the different length of the linkages between the two cavities of **1** and **2**. In addition, thermodynamic results indicate that the complexations of β-CD, **1** and **2** with **3** are driven by entropy. These results confirm that desolvation interaction contribute to the stability of the inclusion.

## Experimental

### General

β-CD was purchased from Sigma-Aldrich Chemical Co. and used without further purification. Compounds **1** [19] and **2** [21] were synthesized and purified as reported in the literature. Compound **3** was prepared as previously described [33]. Disodium hydrogen phosphate and sodium dihydrogen phosphate were dissolved in distilled deionized water to prepare a 0.01 M phosphate buffer solution of pH 7.4 for the UV-Vis spectra measurements. UV-Vis spectra were measured with a Shimadzu 3100 UV-Vis-near-IR recording spectrometer equipped with a temperature controller. The sampling interval was 0.2 nm, and the slit width was at 2.0 mm. The reference cell contained a phosphate buffer solution of the identical host at equimolar concentration. ^1^H-NMR spectra were recorded with a Bruker AM-500 spectrometer (500 MHz) at ambient temperature using D_2_O as solvent. Monodimensional spectra were recorded for (i) **3**, (ii) β-CD, **1** or **2**, (iii) the mixture of **3** and β-CD, **1** or **2**.

### UV-Vis spectra

The UV-Vis spectra of **3** (6 μM) were measured at several temperatures in the presence of varying concentrations of host (β-CD, **1** or **2**) in phosphate buffer solution (pH 7.4). Before the spectra measurement, the mixed solutions of β-CD, **1** or **2** and **3** were sonicated for 3 h in an ultrasonic bath and then incubated for 24 h at a certain temperature. To determine the stability constants (*K*_s_) of the complexes, the spectrophotometric titration method was used. Sample volume was 4 mL. 

### MM2 calculations

The structures of the β-CD, **1**, **2** and **3** were built by using CS Chem3D Ultra, and their geometries were then minimized to a root mean square (RMS) value of 0.01 kcalmol^-1^Å^-1^ with the MM2 force field by using the Polak-Ribiere (conjugate gradient) in vacuum [34]. In the first step, **3** was manually docked within the cavity of β-CD, **1** or **2** through its wider entrance, and then a minimization was carried out with the MM2 force field. After 3,000 conjugate gradient iterations, the constraints were removed and the complex was further minimized [25].
